# *Sinanodonta Woodiana* (Mollusca: Bivalvia: Unionidae): Isolation and Characterization of the First Microsatellite Markers

**DOI:** 10.3390/ijms12085255

**Published:** 2011-08-17

**Authors:** Oana Paula Popa, Luis Ovidiu Popa, Ana-Maria Krapal, Dumitru Murariu, Elena Iulia Iorgu, Marieta Costache

**Affiliations:** 1 Molecular Biology Department, “Grigore Antipa” National Museum of Natural History, Kiseleff Street, No 1, Bucharest 011341, Romania; E-Mails: oppopa@antipa.ro (O.P.P.); popaluis@antipa.ro (L.O.P.); ana.krapal@antipa.ro (A.-M.K.); dmurariu@antipa.ro (D.M.); 2 Departments of Biochemistry and Molecular Biology, University of Bucharest, 91-95 Spl. Independentei, Bucharest 050095, Romania; 3 Faculty of Biology, Alexandru Ioan Cuza University of Iasi, 22, Carol I Boulevard, Iaşi RO 700505, Romania

**Keywords:** invasive species, microsatellite, population genetics, range expansion, native area

## Abstract

*Sinanodonta woodiana* (Lea, 1834) is a large Unionid species with a real invasion success. It colonized Europe, Central America, the Indonesian Islands and recently North America. The species life cycle involves a larval parasitic stage on freshwater fish species which contributes to the spread of the mussel. In this paper we describe, for the first time, eight polymorphic microsatellite loci for the species *Sinanodonta woodiana*. The genetic screening of individuals confirmed that all loci were highly polymorphic. The number of alleles per locus ranged from 7 to 14 and the observed heterozygosity ranged from 0.650 to 0.950. These loci should prove useful to study the species population genetics which could help to infer important aspects of the invasion process.

## 1. Introduction

The freshwater mussel *Sinanodonta woodiana* (Lea, 1834) (Chinese Huge Mussel or Swan Mussel) is one of the largest Unionid species present in the European Fauna. The species is native to South Eastern Asia, specifically Indochina and Southern China to Korea, Japan, Taiwan and the Amur Basin in Eastern Russia [1].

The life cycle of *Sinanodonta woodiana* includes a parasitic larval stage—glochidium—which lives for a short time attached to freshwater fish species. The attached glochidia obtain nourishment from their host [2], but the relationship is more phoretic than nutritive [3].

Across Europe, the spread of the swan mussel was correlated with the introduction in the aquaculture of the East Asian cyprinids species (*Ctenopharyngodon idella*, *Hypophthalmichthys molitrix)* from the Amur River, between 1963–1965 [4,5]. Exports of the host fish species between different countries and regions helped the mussel to penetrate new environments. Physiological, ecological and biological peculiarities of the species offer it some advantages compared with the native unionid species and can explain its invasive success. *S. woodiana* has a particular physiological predisposition, associated with cholinesterase enzyme activity, to tolerate a variety of unsuitable environmental conditions, like pollution and hypoxia, in comparison with the autochthonous species [6]. This is the main cause that allowed the species to colonize Europe in less than 20 years. The expansion of *S. woodiana* is well documented in Europe [5,7–9], Central America [1], Indonesian Islands [10] and recently in North America [11]. In Romania, *S. woodiana* has spread in almost all important aquatic basins [7,12], and it became the prevalent species in the Unionidae communities in terms of biomass, especially in the lower flow of aquatic basins. Studies concerning the population genetics of *S. woodiana* are limited to alozymes analysis of several populations in Poland [13]. No other data are available in the literature about the genetic diversity of *S. woodiana* populations in the native or invaded areas.

Population genetic studies could help to infer important aspects of the invasion process of the species, like the route(s) of invasion, the time and number of colonization events, *etc*., and DNA microsatellite markers are one of the most useful tools to investigate these processes. In this paper we describe, for the first time, eight polymorphic microsatellite loci for the species *Sinanodonta woodiana*.

## 2. Results and Discussion

We selected 95 positive clones (that generated two bands on an agarose gel) by screening the microsatellite enriched genomic library. These were further sequenced using the LICOR 4300L Genetic Analyzer. All of the sequenced clones contained repetitive motifs, but only 30 of the sequenced clones were suitable for primer design. Eight out of 30 primer pairs gave consistent amplification and were subsequently used for polymorphism screening (Table 1). We tested the degree of polymorphism of the isolated loci in 20 individuals of *Sinanodonta woodiana* collected from the Prut River, at Vădeni, Romania. The number of alleles per locus ranged from 7 to 14 and the observed heterozygosity ranged from 0.650 to 0.950 (Table 2). All loci exhibited Hardy-Weinberg equilibrium and no linkage disequilibrium was observed between the markers. The results of the Micro-Checker testing showed no evidence for an excess of homozygotes, or for the presence of null alleles.

The loci described here are important molecular markers that may solve several issues regarding the invasive process of this species, like determining the source populations of invasive species, ways of introduction, initial size of invasive population, sustainability and variability of populations of *S. woodiana*.

## 3. Experimental Section

The microsatellite loci were developed using a modified enrichment protocol [14,15] which was also used to isolate DNA markers for *Hypanis colorata* [16]. Samples of *Sinanodonta woodiana* were collected in 2010 from the Prut River at Vădeni, Romania (46°03′48.44″N, 28°05′16.54″E) and tissue samples were preserved in 95% ethanol.

The PCR primers for each microsatellite locus were designed with the Primer3 program [17]. The PCR genotyping reaction was performed in a 10 μL total volume containing about 50 ng of DNA template, 10 mM Tris-HCl (pH 8.8 at 25 °C), 50 mM KCl, 0.08% (v/v) Nonidet P40, 2.5 mM MgCl_2_, (see Table 1 for details for each locus), each dNTP at 0.1 mM, each primer at 0.1 μM, 0.02 μM of IRD700 or IRD800 labeled M13 primer and 0.5 units of Taq DNA polymerase (Fermentas UAB, Vilnius, Lithuania). The temperature profile of the PCR reaction consisted of an initial denaturation step at 95 °C for 3 min followed by 30 cycles of denaturation at 95 °C for 30 s, annealing at a specific temperature for each locus (see Table 1 for details for each locus) for 30 s and extension at 72 °C for 45 s, followed by a final extension step at 72 °C for 5 min. The genotyping process was performed using SagaGT ver. 3.1 software package.

GenAlEx 6.4 [18] was used to estimate the number of alleles per locus (N_a_), observed heterozygosity (H_obs_) and expected heterozygosity (H_exp_). Deviation from the Hardy-Weinberg equilibrium (HWE) was tested using the same software package. The presence of null alleles was tested using Micro-Checker (ver. 2.2.3) [19] while linkage disequilibrium test was carried out using Arlequin ver. 3.1 [20].

## 4. Conclusions

In the present study we described eight polymorphic microsatellite loci for the highly invasive Asian mussel *Sinanodonta woodiana*. These loci would be useful to determine the source populations of this invasive species, potential ways of introduction, the number of colonization events, initial size of the established population and variability of populations of *S. woodiana* in native and invaded areas.

## Figures and Tables

**Table 1.** Primer sequences and characteristics of eight microsatellite loci successfully amplified in *Sinanodonta woodiana*. Ta, annealing temperature; [MgCl_2_], MgCl_2_ concentration in the PCR reaction; Size, size range of alleles.

**Marker**	**Genebank Accession number**	**Repeat motif**	**Primer sequence**	**Ta**	**[MgCl_2_]**	**Size**
SW2	JN180655	CA	F: caaaaatgaaccggacacct	51	2.5	152–194
			R: cccaaactcgttttcattgg			
SW3	JN180656	AT	F: tgaaactggtgtccaatcca	53	2.5	154–184
			R: ctcccgaaagagcacaacat			
SW4	JN180657	TG	F: agcgcaattaccagtggttt	51	2.5	215–229
			R: ttgattgcgatgactggaaa			
SW7	JN180658	CA	F: ctgccacactgcagatattgt	51	2.5	213–233
			R: aacacgttcgaaatccgagt			
SW13	JN180659	AC	F: cgccatgtcaaaaatcaaag	55	2.5	237–283
			R: gccacaagcatgagatgtgt			
SW14	JN180660	TG	F: cccgtgttgtcaaaggaaat	53	2.5	178–222
			R: tttttctggcgactttccac			
SW15	JN180661	CA	F: aaccgacaagtccttgcaata	53	2.5	307–345
			R:cagctgagtcgattaggacaga			
SW18	JN180662	GTTT	F: gtaacgtctcgtcgggtcat	53	2.5	142–220
			R: gcctcggtgctagacatcac			

**Table 2.** Variation across 8 microsatellite loci in a population of 20 *Sinanodonta woodiana* individuals. N_a_: number of alleles; H_obs_: observed heterozygosity; H_exp_: expected heterozygosity; HWE-p: p- value of chi square test for Hardy-Weinberg equilibrium.

**Marker**	**N_a_**	**H_obs_**	**H_exp_**	**HWE-p**
SW2	14	0.950	0.874	0.245
SW3	8	0.650	0.710	0.338
SW4	7	0.700	0.765	0.104
SW7	8	0.750	0.791	0.523
SW13	11	0.800	0.841	0.248
SW14	11	0.900	0.864	0.810
SW15	10	0.900	0.859	0.273
SW18	9	0.950	0.849	0.176

## References

[1] Watters TG (1997). A synthesis and review of the expanding range of the Asian freshwater mussel Anodonta woodiana (Lea, 1834) (Bivalvia:Unionidae). Veliger.

[2] Wachtler K, Dreher-Mansur MC, Richter T, Bauer G, Wachtler K (2001). Larval Types and Early Postlarval Biology in Naiads (Unionoida). Ecology and Ecolution of Freshwater Mussels Unionoida.

[3] Barnhart MC, Haag WR, Roston WN (2008). Adaptation to host infection and larval parasitism in Unionida. J. N. Am. Benthol. Soc.

[4] Petro E (1984). The occurance of Anodonta woodiana woodiana in Hungary. Állaltani Közl.

[5] Sarkany-Kiss A (1986). Anodonta woodiana (Lea, 1834) a new species in Romania (Bivalvia, Unionacea). Trav Mus Hist Nat Grigore Antipa.

[6] Corsi I, Pastore AM, Lodde A, Palmerini E, Castagnolo L, Focardi S (2007). Potential role of cholinesterases in the invasive capacity of the freshwater bivalve, *Anodonta woodiana* (Bivalvia:Unionacea): a comparative study with the indigenous species of the genus *Anodonta* sp. Comp. Biochem. Phys. C.

[7] Popa O-P, Kelemen SB, Murariu D, Popa LO (2007). New records of Sinanodonta woodiana (Lea, 1834) (Mollusca: Bivalvia) from Eastern Romania. Aquat. Invasions.

[8] Paunovic M, Csanyi B, Simic V, Stojanovic B, Predrag C (2006). Distribution of *Anodonta (Sinanodonta) woodiana* (Lea, 1834) in inland waters of Serbia. Aquat. Invasions.

[9] Cappelletti C, Cianfanelli S, Beltrami ME, Ciutti F (2009). Sinanodonta woodiana (Lea, 1834) (Bivalvia:Unionidae): a new non-indigenous species in Lake Garda (Italy). Aquat. Invasions.

[10] Djajasasmita M (1982). The occurrence of Anodonta woodiana Lea, 1837 in Indonesia (Pelecypoda, Unionidae). Veliger.

[11] Benson AJ Sinanodonta woodiana. http://nas.er.usgs.gov/queries/FactSheet.aspx?speciesID=2824.

[12] Popa OP, Popa LO (2006). *Sinanodonta woodiana* (Lea, 1834), *Corbicula fluminea* (O.F.Muller, 1774) and *Dreissena bugensis* (Andrusov, 1897) (Mollusca:Bivalvia): Alien invasive species in Romanian Fauna. Trav. Mus. Hist. Nat. Grigore Antipa.

[13] Soroka M (2005). Genetic variability among freshwater mussel *Anodonta woodiana* (Lea, 1834) (Bivalvia:Unionidae) populations recently introduced in Poland. Zool. Sci.

[14] Bloor PA, Barker FS, Watts PC, Noyes HA, Kemp SJ Microsatellite Libraries by Enrichment. http://www.genomics.liv.ac.uk/animal/research/microsatellite.pdf.

[15] Carleton KL, Streelman JT, Lee BY, Garnhart N, Kidd M, Kocher TD (2002). Rapid isolation of CA microsatellites from the tilapia genome. Anim. Genet.

[16] Popa OP, Iorgu EI, Krapal AM, Kelemen SB, Murariu D, Popa LO (2011). Isolation and Characterization of the First Microsatellite Markers for the Endangered Relict Mussel Hypanis colorata (Mollusca: Bivalvia: Cardiidae). Int. J. Mol. Sci.

[17] Rozen S, Skaletsky H, Krawetz S, Misener S (2000). Primer3 on the WWW for General Users and for Biologist Programmers. Bioinformatics Methods and Protocols.

[18] Peakall R, Smouse PE (2006). GENALEX 6: Genetic analysis in Excel. Population genetic software for teaching and research. Mol. Ecol. Notes.

[19] Van Oosterhout C, Hutchinson WF, Wills DPM, Shipley P (2004). Micro-checker: Software for identifying and correcting genotyping errors in microsatellite data. Mol. Ecol. Notes.

[20] Excoffier L, Laval G, Schneider S (2005). Arlequin ver. 3.0: An integrated software package for population genetics data analysis. Evol. Bioinforma. Online.

